# Analysis of the response subsidence and grouting treatment in the goaf of multi-layer inclined coal seam

**DOI:** 10.1038/s41598-023-47909-9

**Published:** 2023-11-24

**Authors:** Xuhe Gao, Weiping Tian, Qinggang Pang, Jiachun Li, Hongliang Qi, Bin Xu, Zhipei Zhang

**Affiliations:** 1 School of Mechanics, Civil Engineering and Architecture, Northwestern Polytechnical University, No. 1 Dongxiang Road, Chang’an District, Xi’an, 710129 Shaanxi China; 2 Observation and Research Station of Ground Fissure and Land Subsidence, Ministry of Natural Resources, Xi’an, Shaanxi, No. 100 Yanta North Road, Beilin District, Xi’an, 710054 Shaanxi China; 3 Key Laboratory of Highway Engineering in Special Region, Ministry of Education, Chang’an University, No. 126, Middle Section of South Second Ring Road, Beilin District, Xi’an, 710064 Shaanxi China; 4 College of Geology and Environment, Xi’an University of Science and Technology, No. 58 Yanta Middle Road, Beilin District, Xi’an, 710054 Shaanxi China; 5 Capital Engineering & Research Incorporation Limited, China Metallurgical Group Corporation, No. 7 Jian’an Street, Beijing Economic and Technological Development Zone, Beijing, 100176 China

**Keywords:** Natural hazards, Civil engineering

## Abstract

Based on existing researches, field drillings and numerical simulations are carried out in this paper to analyze the problems of subsidence control in the goaf of multi-layer inclined coal seam. Midas/GTS NX is used to build a three-dimensional calculation model of the goaf. A new method of using borehole data to check simulation parameters is proposed. The whole process of goaf excavation, construction of roadbed (pile foundation) and grouting treatment is analyzed. Analysis theory of different subgrade construction schemes and grouting treatment process on goaf is established. Response characteristics of displacement and equivalent stress and strain of goaf in multilayer inclined coal seam are obtained. A new method for analyzing the characteristics of the stress and deformation of the rock strata before and after grouting in the goaf under the conditions of different foundation schemes on the surface is provided in this research.

## Introduction

The area where the surrounding rock instability of the underground mining space leads to the overall subsidence and bending of the overlying strata. The area caused by surface deformation and damage is called a goaf. The instability leads to sinking and bending of the overlying rock layer, which causes surface deformation and damage. When a highway or other surface structure is built over the mined-out area, also known as a goaf site, the ongoing subsidence of the goaf foundation may be exacerbated in the long-term effects of the roadbed or other foundational load. A previously stable goaf foundation may be destabilized by the surface load. Therefore, choosing a suitable foundation structure and determining a effective grouting treatment is critical for any construction on the surface of the goaf.

Many scholars have examined the ground subsidence caused by coal mining with on-site measurement, theoretical analysis, numerical simulation and physical similarity simulation tests^1–6^. Physical and numerical simulation methods were used by Xu et al.^7^ to study the impact of key overburdened strata on the dynamics of ground subsidence. Yin et al.^8^ combined similar model tests and numerical analysis to investigate the characteristics of mining rock movement, mine pressure distributions, surface movement and deformation in the context of deep incline coal mining. Huang et al.^9^ used a combination of physical simulation, numerical calculation and field measurement to study the evolution and mechanism of overlying strata and surface fissures in a shallow coal seam mining. Sun et al.^10^ used numerical simulation and similarity simulation experiments to study the effects of double seam mining on overlying strata. Research shows that the greater the width of the coal pillars left in the working face is, the less the impact of coal seam mining on the overlying strata is. Existing researches mainly focus on theoretical analysis, similar simulation experiments and comparative verification of numerical simulation results^11–15^. The mining subsidence and grouting treatment of multi-layer inclined coal seam based on field drilling data to validate simulation parameters are not involved.

The finite element method, the finite difference method and the discrete element method are the common methods for numerical simulation of overburdened mined-out areas and the movement and deformation of the ground surface. J. Wang et al.^16^ used FLAC3D numerical simulation software to establish a three-dimensional multi-layer, wide and gentle fold geological model. The vertical displacement and the tunnel radial and tangential stress of the study area were obtained in numeric simulations. MIDAS/GTS finite element software was adopted by Han et al.^17^ to simulate the subsidence and safety of a mining road near a subsidence area during underground mining. Theoretical data for the effective management of the subsidence area was obtained. Gao & Chen^18^ used the discrete element software CDEM based on continuum mechanics to study the distribution and deformation law of the "three zones" of the goaf.

In terms of grouting control, with the widespread of computer technology, researchers are committed to the computerization of "three-under" mining subsidence prediction and grouting control^19–29^. The underground mined-out area of the Taijia Expressway was divided into three geological forms by Liu^30^: surrounding rock slump accumulation area, coal pillar and cavity. Applying the theory of permeation grouting to the infiltration of slurry in the rock slump accumulation area in the goaf, the slurry diffusion characteristics and laws were provided. Both the goaf grouting reinforcement mechanism and strip-mining theory were adopted by Li et al.^31^ to create a model and study the applications and critical parameters of the strip grouting method for controlling the residual subsidence of goaf. The numerical simulation was used to compare and analyze the subsidence deformation and vertical stress distribution of the foundation before and after belt grouting and after the load is applied.

Theoretical analysis performed is insufficient for the process analysis of subsidence and goaf treatment in a multi-layer inclined coal seam. However, traditional numerical simulation parameter determination methods need more validation. A case study of grouting treatment in a multi-layer inclined coal seam is performed in this work. Based on preliminary identification of the stratum structure through on-site drilling, Midas/GTS NX software is used to establish a three-dimensional calculation model. A new method for simulation parameter validation and process analysis of the goaf is proposed. The force and deformation characteristics of the goaf are investigated in three conditions: mining completion, roadbed/pile foundation construction, and goaf grouting. Comparative analysis of the roadbed scheme and the pile foundation scheme for surface structure construction are also performed. Theoretical support is provided for roadbed selection and grouting treatment evaluation for highway structures above a mined-out area.

## Materials and methods

### Geological overview

The research area is in Nanguanzhuang, Gongyi City, Henan Province. The Jiaotong Expressway passes through this area as a roadbed. Located at the junction of the Yellow River and the Huai River, the surface runoff and inflow water mainly come from natural precipitation that is mostly concentrated during the flood season. The main strata in the area are Quaternary alluvial soil, gravel soil, Carboniferous Permian sandstone, and Ordovician limestone.

The mining area is in the Songji structural area in the southern part of the North China Plate. It belongs to the north wing of the Songshan anticline and is located on the footwall of the Wuzhiling fault. The overall structure is a monoclinic structure. The rock formation strike in the study area is 50°, inclination 320°, and dip angle 35–37°. The upper part of the coal seam is about 69 m away from sandstone, and the lower part is about 11 m away from limestone, with a thickness of 6.50–15.00 m. The roof is dominated by medium-fine grained long quartz sandstone, and the indirect floor is mostly banded sandy mudstone. In the study area, the recovery rate of coal mining face is 95%. The regularity of the extension direction of the surface cracks is not obvious.

### Core analysis

14 boreholes (including three repetitive boreholes) are arranged in and around the mining area with a total drilling footage at 2,331.15 m, a geophysical survey area of 210,000 m^2^, 12 IP sounding profiles, a survey line length of 3645 m, a survey line direction of 165°, a mesh size of 60 m × 30 m, and in total of 125 sounding physical points.

**Figure 1:**
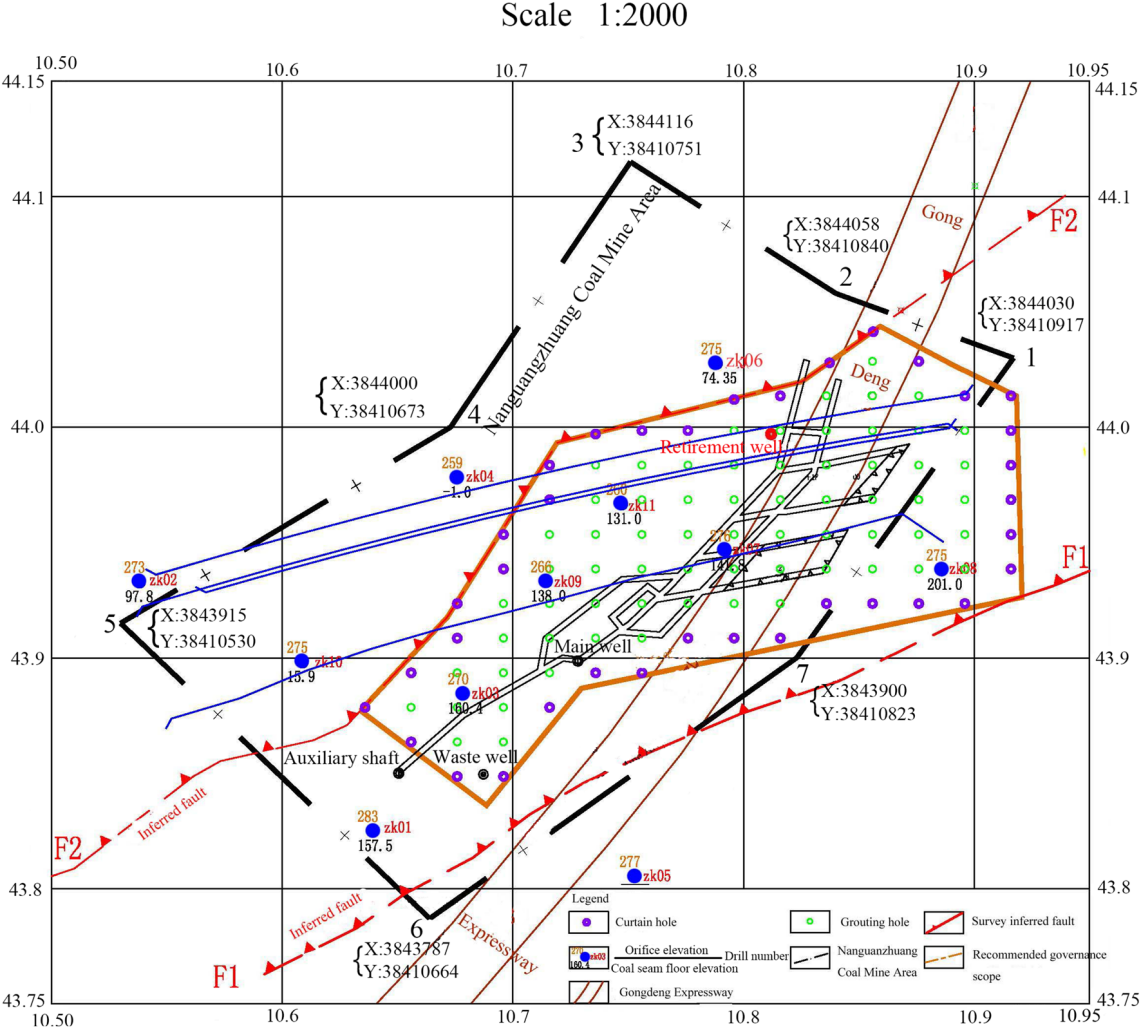
Schematic of the mined-out area landmarks.

**Figure 2:**
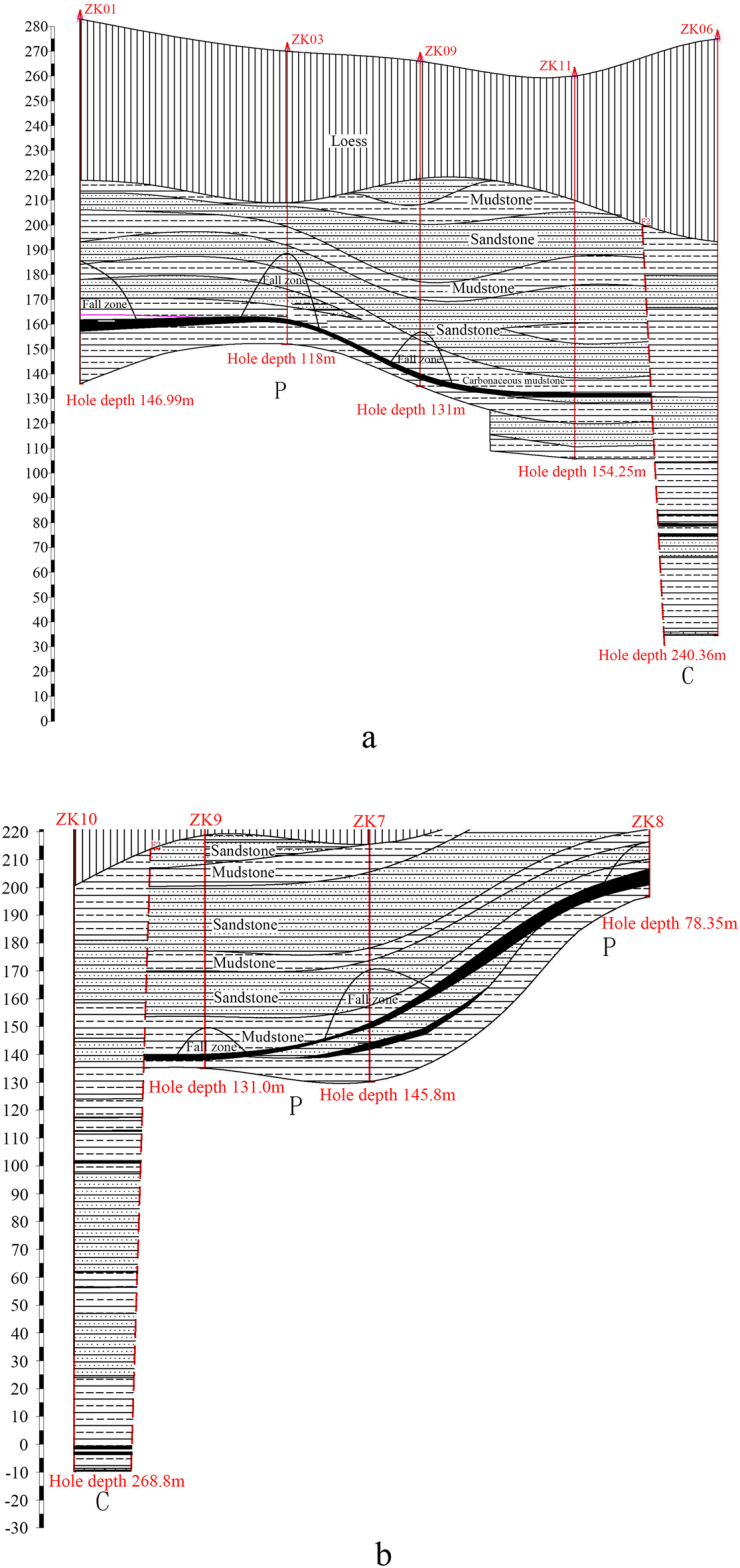
Stratum cross-section as revealed by drilling.

Through geophysical prospecting and drilling, the hidden Quaternary and Permian soil-rock interfaces are identified, as well as the locations of the F1 and F2 faults in the exploration area. The drilling positions are shown in Fig. 1. The drilled holes reveal the cross-section, as shown in Fig. 2.

Drilling data and geophysical prospecting results show that: No. 6 and No. 7 boreholes are in a multi-layer inclined coal seam. Meanwhile, the stratum features of No. 6 and No. 7 boreholes are obvious. The specific data are as follows:

Drilling hole No. 6 is located to the north of the F_2_ fault. Total thickness of the Quaternary loess, pebble and boulder layer is 81.7 m. The hole “zk6” was drilled to 240.36 m deep at a secondary location and chemical tests determined that the core was limestone. There are 4 layers of coal at 170.6–171.0 m, 191.9–192.25 m, 195.6–196.55 m, and 200.0–200.65 m deep in the hole, with a thickness between 0.325 and 0.95 m. This is an unminable coal seam.

Drilling hole No. 7 is located between the F_1_ and F_2_ faults. The thickness of the Quaternary loess and pebble layer is 60.5 m. During the drilling of the No. 7 borehole, water leaked at 70.5 m deep, with additional serious water leakage at 102.3–124.8 m deep. A coal seam was found at 124.8–126.6 m and 132.2–134.2 m deep. The drilling hole is 145.8 m deep, and the final hole is in Permian mudstone.

The Nanguanzhuang Coal Mine was built in 1985. The original main shaft was scrapped due to flooding. The mine has a pair of main and auxiliary shafts, with a designed production scale of 90,000 tons per year, and a coal mining thickness of 6.5 to 15 m. The mine uses the strike long arm retreat mining and the total subsidence method for roof management. The mine has been suspended since the end of 2005.

According to the investigation of the subsidence and penetration of the mined-out area, it is planned to determine the grouting hole mesh size to be 15 × 20m. There are 69 grouting holes and 35 curtain grouting holes in the whole area, with a grouting volume of 8500 m^3^. The specific grouting sequence and actions are as follows: Firstly, a water stop curtain is formed by grouting through the curtain grouting hole to prevent the entry of groundwater from the external area and the subsequent outflow of grout from the internal area (purple hole in Fig. 1). Secondly, the goaf is filled with grouting through grouting holes (green holes in Fig. 1).

The drilling rig is drilled in place, and a grout stopper is placed in the hole, and the bottom of the hole is returned to the layered grouting process. When grouting for the first time, the working pressure is controlled at about 0.5MPa, and the grouting flow rate is controlled at 50L/min. When grouting, the slurry is insured to rise evenly until thick slurry emerges from the orifice. Each time 2m is to be pulled out, it must be refilled in time to ensure the continuity of the entire grouting process.

### Calculation of overlying rock subsidence

The maximum surface subsidence of the goaf was determined as follows^32^:

1 $${W}_{max}=M\cdot q/cos\alpha ,$$

where W_max_ is the maximum subsidence of the ground surface (in meters), M is the normal thickness of the inclined ore bed (in meters), α is the mineral layer inclination angle, and q is the sink coefficient. In the study area, M = 1.6 m (The average coal seam thickness of blue boreholes 3, 6, 7, 8, 9, and 11 as shown in Fig. 1.), α = 15°, and q = 0.85 (Approximate value; the value is obtained by referring to Table D.0.1–1 in Guidelines for Design and Construction of Highway Engineering in the Mined-out Area (JTG/T D31-03-2011) according to rock conditions). Thus, W_max_, the accumulated maximum surface subsidence of multi-layer inclined coal seams, is 1.408 m.

## Modeling and methods

### Simulation assumptions

Midas/GTS NX software is used for modeling. Considering the limitations of regional mining history, geological conditions and the grouting construction environment, the following assumptions were made for the model:

1. The rock-soil mass is an ideal elastoplastic body and obeys the strength yield criterion of Mohr–Coulomb.
2. The influence of groundwater and seepage on the model can be simplified.
3. The structural unit of the pile foundation is a completely elastic body, and an elastic constitutive model can be adopted. The rock-soil-structure interface is defined by the pile contact characteristics.

**Figure 3:**
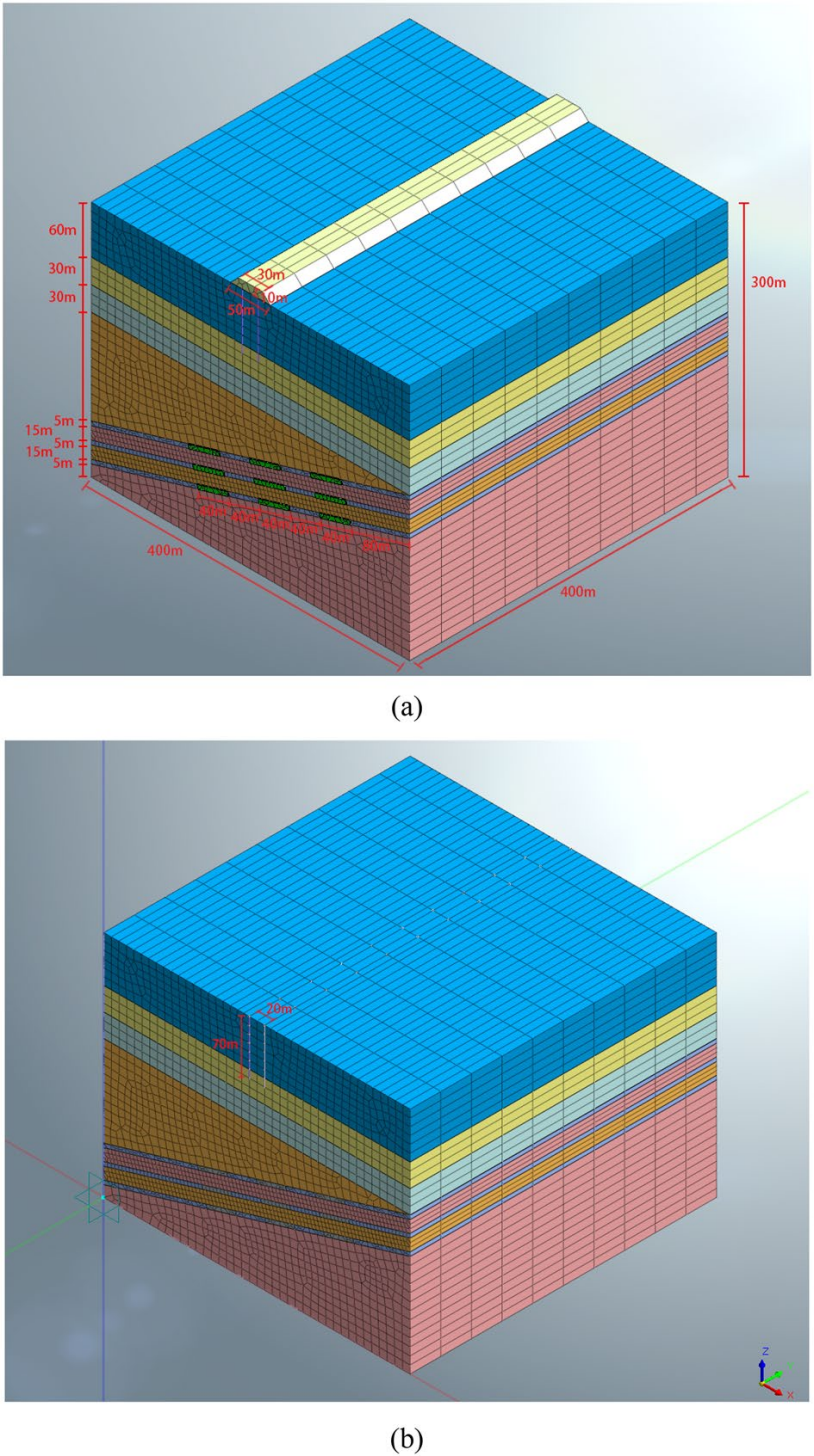
Calculation model for the (**a**) roadbed with subgrade scheme and (**b**) pile foundation scheme.

According to the cross-section revealed by the borehole data and the preliminary research needs, a three-dimensional model with length, width, and height of 400 × 400 × 300 m (Fig. 3) is constructed.

From the surface down, the geological layers of the model are loess, sandstone1, mudstone1, sandstone2, coal seam, mudstone2, coal seam, sandstone2, coal seam, and mudstone 2 (Fig. 3a). The layer thicknesses are 60 m (loess), 30 m (first sandstone), 30 m (mudstone), and 180 m (the total thickness of sandstone, coal seam, and mudstone, assuming that the coal seam is 5 m thick and the spacing is 15 m).

The first layer of coal is inclined 15° downward from the edge of the model, with a thickness of 5 m. The coal seams are mined at an interval of 40 m to form a goaf. In the roadbed scheme, the thickness of the subgrade is 10 m, the width of the subgrade bottom is 50 m, and the width of the subgrade top is 30 m. In the bridge scheme, the pile foundation is buried 70 m into the ground (Fig. 3b). The force of the single pile is 20,000 kN, and the pile diameter is D = 1 m. Suppose the piles are spaced 40m apart along the route.The centerline of the roadbed and pile foundation are both located in the plane of the model (200, Y, Z). The specific dimensions of the model are shown in Fig. 3a and b.

The model default contact parameters: the normal stiffness scaling factor is 1, and the tangential stiffness scaling factor is 0.1. In the mean time, the auxiliary nodes are adjusted to eliminate internal penetration. The geotechnical layer is controlled by entity attributes in the model. The grid is divided into 10m in the XZ plane and 40m in the Y direction with equal thickness. The pile is controlled by line properties in the model, and the diameter of the section is 1.0m. The reinforced concrete constituting the pile has a thermal expansion coefficient of 1e − 006 and a damping ratio of 0.05. The grouting simulation is realized by filling the mined area with grout material.

#### Model boundary conditions

The model boundary conditions are as follows.

1. The X-direction of the left and right boundaries of the model is constrained and u = 0, where u is the displacement in the X-direction, v is the displacement in the Y-direction, and w is the displacement in the Z-direction.
2. The front and back boundaries of the model are constrained in the Y-direction and v = 0.
3. The bottom boundary of the model is fully constrained, with u = 0, v = 0, and w = 0.
4. The upper boundary of the model is defined as a free boundary, and no constraints are given.

The initial stress is the self-weight stress of the formation. A model of 400 × 400 × 300 m is established based on the assumptions above. The roadbed scheme contains 22,407 units and 61,926 calculation equations; the pile foundation scheme includes 22,319 units and 62,206 calculation equations.

#### Model parameter validation

The simulation calculations for the goaf are affected by several key geotechnical parameters. The current methods for parameter selection and determination include geotechnical engineering laboratory tests, use of statistical data, and empirical data based on similar geological features. However, there are issues of non in-situ and disturbance in indoor geotechnical engineering tests, which may not be consistent with the actual engineering situation. Furthermore, the use of statistical data is only applicable to ordinary stratum and requires the accumulation of extensive engineering knowledge. Finally, although empirical data is convenient to use, it is lacking in rigor. The three existing methods share a critical disadvantage with regards to modelling goaf in a complex geological environment: the parameter selection method is not universal. Therefore, in traditional theoretical calculations and finite element analysis, it is important to use field drilling data to validate the main model parameters.

Deviation rate is a statistical indicator which is used for comparing actual measurements with theoretical benchmarks. It can be used to test whether a certain production or service process can be effectively controlled. The calculation formula for deviation rate is deviation rate = (actual value—standard value)/standard value × 100%. The deviation rate can be limited to 1%, 3%, 5%, 10%, 15%, etc. in different industries. A deviation rate limit of 10% is selected based on the verification needs of multiple working conditions and comprehensive analysis of on-site investigations.

In response to the contradiction in selecting simulation parameters, the concept of the deviation rate between simulated values and theoretical calculated values in introduced in this study. A simulation parameter verification method based on on-site drilling data, namely key data self-verification method, has been proposed. In biref, when the basis for parameter selection is not convincing enough, the initial parameters are used for trial and iteration of the simulation results. When the deviation rate P between simulated data and actual measurement is less than 10%, the physical and mechanical parameters used in the calculation model are valid. After validating the simulation parameters, further analysis could be performed (Fig. 5). Under the Mohr–Coulomb elastic–plastic quasi-conditions, the cohesion (c), internal friction angle (φ) and elastic modulus (E) of the rock mass have the greatest influence on the calculation results^33, 34^. Therefore, c, φ and E are selected as the checking objects of this study (Table 1 Initial Values).

**Table 1:** Initial value of geotechnical parameters.

Layer #	Rock and soil	Elastic modulus (MPa)	Cohesion (KPa)	Friction angle (°)
1	Roadbed	500	100	30
2	Loess	100	30	20
3	Sandstone1	800	500	20
4	Mudstone1	800	500	20
5	Coal Seam (× 3)	500	200	30
6	Sandstone2	800	500	30
7	Mudstone2	800	500	30
8	Slurry	400	50	25

The values in this table are recommended based on existing research data and on-site geotechnical tests. The on-site drilling revealed that the multiple layers of horizontal sandstone and mudstone above the upper coal seam are located in close proximity and present a dogtooth interlocking state. The modeling here simplifies the multi-layer mudstone and sandstone far from the upper coal seam into mudstone 1 and sandstone 1.

**Figure 4:**
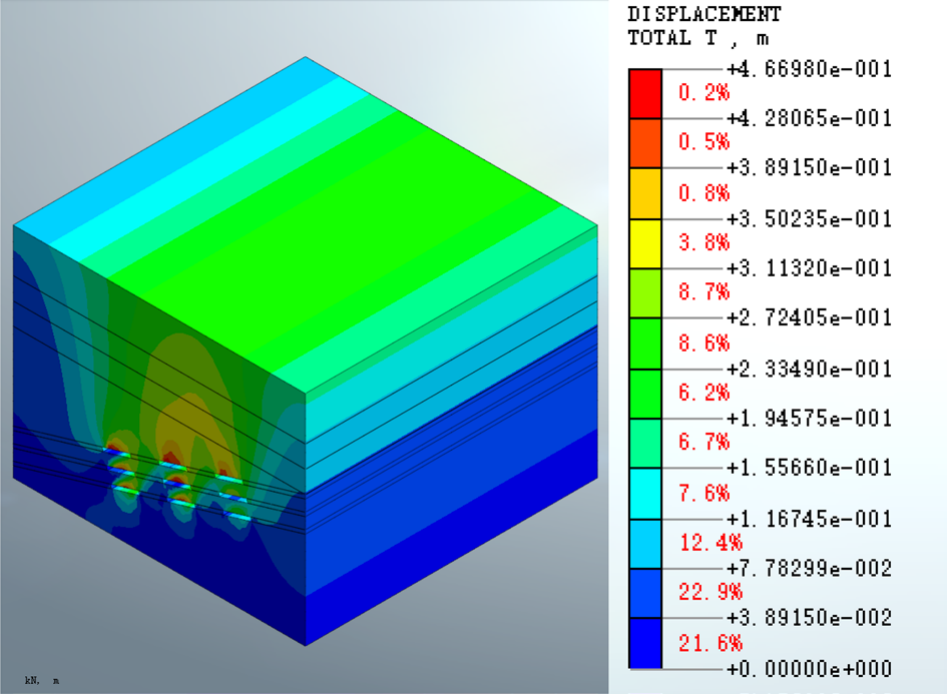
Total displacement of the mined-out area for the excavation completed condition.

The distribution coefficient n is defined as the ratio of the maximum displacement of the roof of a certain coal seam to the sum of the maximum displacements of the three-layer coal roof. According to the calculated total displacement of the coal seam after excavation (Fig. 4), the maximum displacement of the roof of the three coal seams is as follows: top roof, h_1_ = 0.428 m; middle roof, h_2_ = 0.467 m; bottom roof, h_3_ = 0.428 m. The distribution coefficient n for the same structures are as follows: n_1_ = 0.428/(0.428 + 0.467 + 0.428) = 32.35%; n_2_ = 0.467/(0.428 + 0.467 + 0.428) = 35.30%; n_3_ = 0.428/(0.428 + 0.467 + 0.428) = 32.35%.

The surface subsidence h = 1.408 m that may be caused by the subsidence of the coal seam was determined from the drilling data. Distribution coefficient is used to obtain the actual roof subsidence of the multi-layer inclined coal seam. The resultant subsidence of the top roof is H_1_ = hn_1_ = 0.455 m, the middle roof is H_2_ = hn_2_ = 0.498 m, and the bottom roof is H_3_ = hn_3_ = 0.455 m.

Based on the equation for the deviation rate such that

$$\text{P=(analog value-calculated value of drilling data)/calculated value of drilling data,}$$

P_1_ = (h_1_-H_1_)/H_1_ = 5.9%, P_2_ = (h_2_-H_2_)/H_2_ = 6.2%, P_3_ = (h_3_-H_3_)/H_3_ = 5.9%. P_1_, P_2_, and P_3_ all meet the criteria P < 10%. Until now, effective simulation parameters for the calculation and analysis of the next working condition are obtained, otherwise the trial calculation will be conducted again.

#### Process analysis

**Figure 5:**
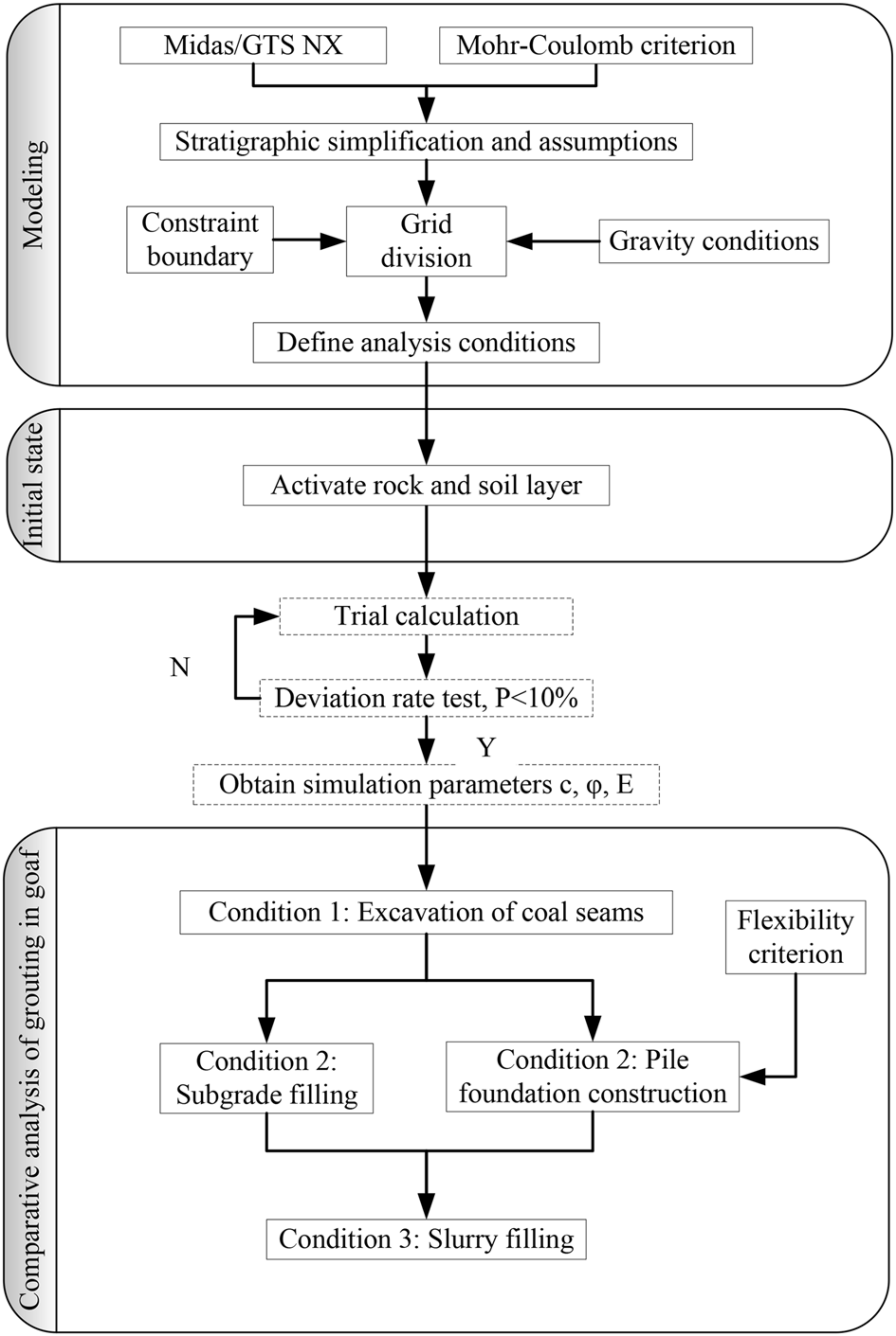
Process analysis flow chart.

The subsidence of the mined-out area proceeds slowly as the forces and deformation accumulate. The subsidence and deformation of the goaf is not a problem of process instead of state. To examine the deformation and failure of the rock structure in the process of excavating coal seams, building upper structures and performing grouting treatment, a process analysis method is proposed. Specifically, we analyzed the model under three conditions: the completion of the excavation of the goaf in the multi-layer inclined coal seam (working condition 1), the construction of the roadbed or pile foundation (working condition 2), and the grouting of the goaf (working condition 3). The calculation process is shown in Fig. 5.

## Results and discussion

### Physical and mechanical parameters

The bulk weight and poisson's ratio in the physical and mechanical parameters of the geotechnical layer are obtained based on field drilling and geotechnical tests. Elastic Modulus, cohesion and friction angle were determined using key data self-validation methods. The specific parameters for each layer are shown in Table 2.

**Table 2:** Physical and mechanical parameters of each rock and soil layer.

Layer #	Rock and soil	Thickness (m)	Bulk weight (kN/m^3^)	Poisson's ratio (1)	Elastic modulus (MPa)	Cohesion (KPa)	Friction angle (°)
1	Roadbed	10	21.5	0.30	400	98	31
2	Loess	60	17.8	0.40	90	23	25
3	Sandstone1	30	21.0	0.30	600	300	25
4	Mudstone1	30	22.0	0.30	600	300	25
5	Coal Seam (× 3)	5	20.0	0.30	600	300	28
6	Sandstone2	–	21.0	0.30	1000	600	28
7	Mudstone2	–	22.0	0.30	1000	600	28
8	Slurry	5*3	20.0	0.40	500	52	25
9	Pile Foundation	–	23.5	0.15	27,930	–	–

### Simulation calculation result analysis

The total displacement can reflect the cumulative position change of each element calculated by modeling. The equivalent stress represents the value of the model calculation element under the Mohr–Coulomb elastoplastic yield criterion. The equivalent strain is the strain equivalent to the unidirectional strain formed by the combination of the strain components of the element under the yield criterion. Three parameters are adopted in analyzing the calculation results, total displacement, equivalent stress and equivalent strain. The stress–strain nephotogram shows positive tension and negative compression.

#### Total displacement

Analysis of Fig. 4 shows that the maximum displacement of the mined-out area following the completion of excavation is 4.66980e − 1 m. The displacement happens at the top of the roof near the empty surface of the hollow in the middle of the upper mined-out area. The displacement of the three-layer goaf shows an obvious superimposition effect, and the maximum displacement of the top layer of the goaf is greater than that of the lower layers. A continuous distribution zone of upward arch-type displacement is evident in the upper part of the top layer of the goaf, and the displacement extends from the roof of the top layer to the surface. The subsidence in the middle of mined-out area is greater than that at the edge.

**Figure 6:**
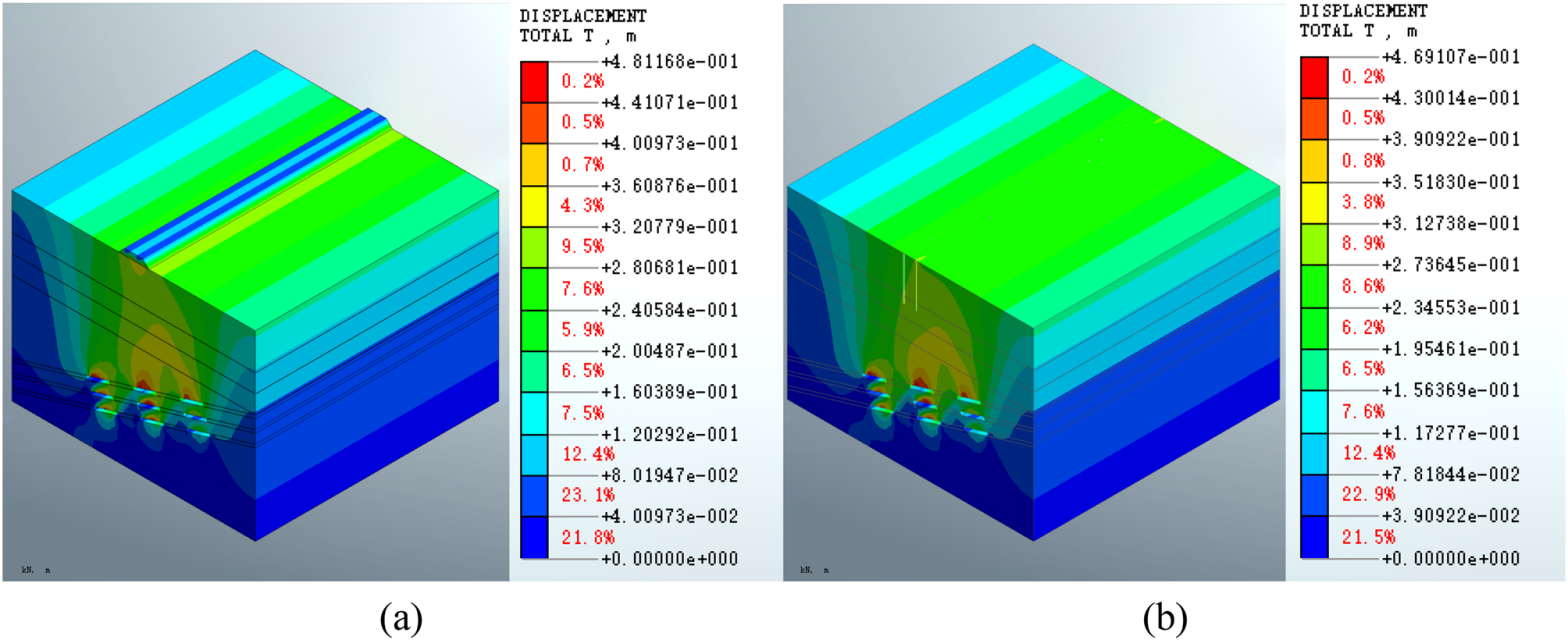
Total displacement of the mined-out area following the construction of (**a**) a roadbed subgrade or (**b**) a pile foundation.

Analysis of Fig. 6 shows that the maximum displacement of the mined-out area in the roadbed scheme is 4.81168e − 1 m (Fig. 6a), which increases compared to the completion of excavation. The maximum displacement of the mined-out area in the pile foundation scheme increases to 4.69107e − 1 m (Fig. 6b). The displacement distribution patterns of the two schemes are close. At the surface, the roadbed construction scheme leads to an increase in the displacement of the middle part of the ground surface; however, this feature is not observed in the pile foundation scheme.

**Figure 7:**
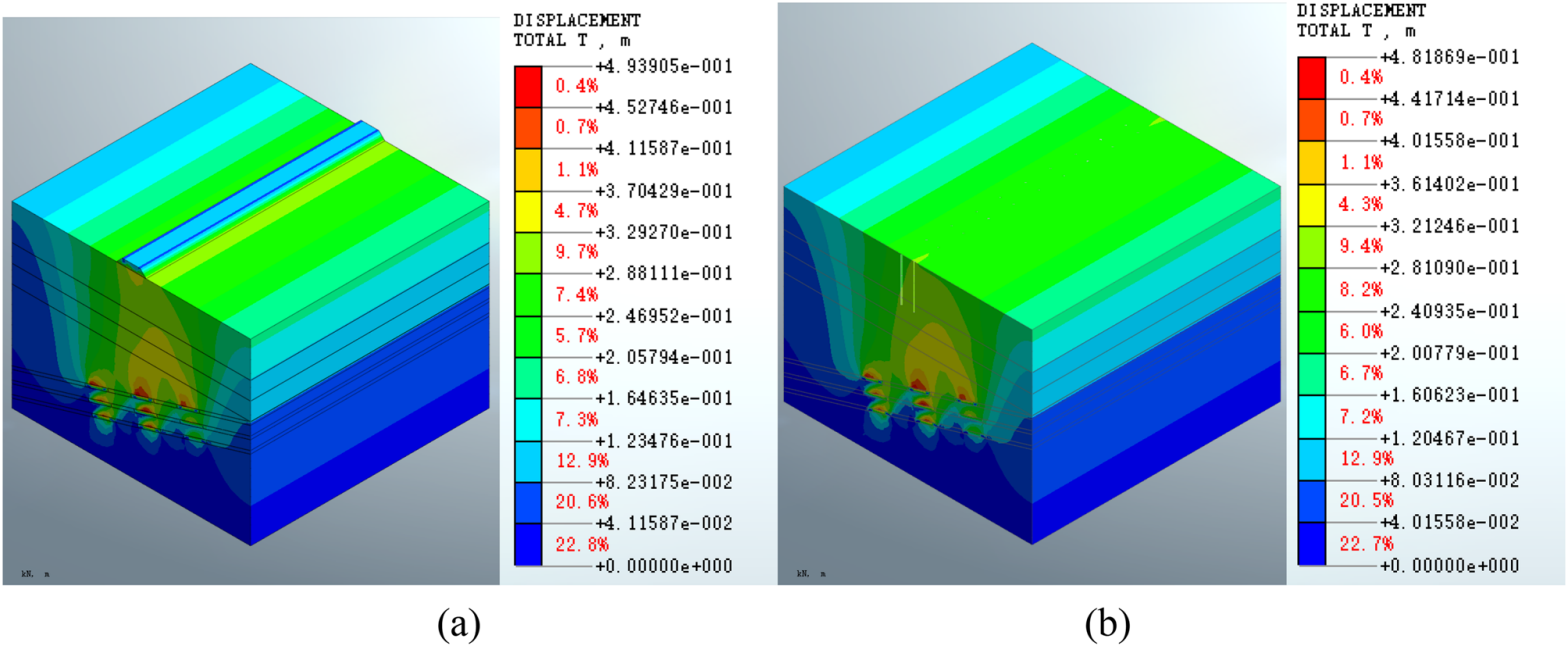
The total displacement of the mined-out area for the (**a**) roadbed scheme and (**b**) pile foundation scheme following grouting treatment.

Analysis of Fig. 7 shows that following grouting treatment, the maximum displacement of the mined-out area is 4.93905e − 1 m (Fig. 7a) for the roadbed scheme and 4.81869 e − 1 m (Fig. 7b) for the pile foundation scheme. The displacement distribution is similar between the two schemes in the deep part of the formation. The displacement distribution characteristics are also close to those described above.

The displacement results show that the displacement distribution patterns in the mined-out area are similar for both the roadbed and the pile foundation schemes. The maximum displacement is increased by 3.04% in construction of the roadbed subgrade (in the roadbed scheme) and by 0.46% in pile foundation construction. Compared the post-construction with post-grouting conditions, it shows disturbance of the grouting will lead to increasing displacement near the goaf. Calculation of the displacement index shows that the pile foundation scheme has less impact on the mined-out area.

#### Equivalent stress

**Figure 8:**
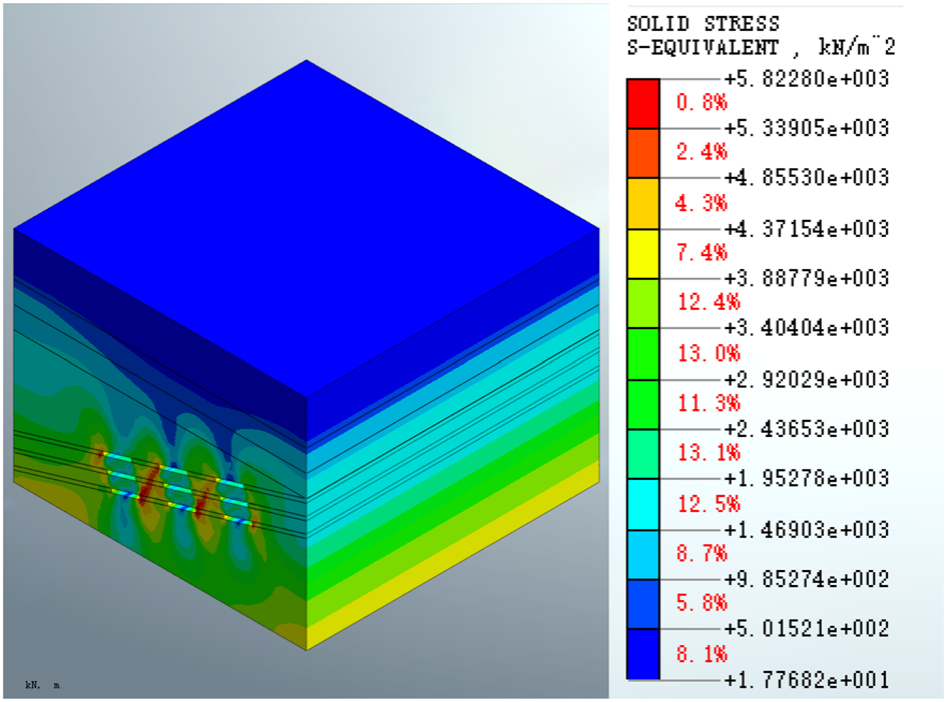
Equivalent stress on the mined-out area following excavation.

Analysis of Fig. 8 shows that the maximum equivalent stress on the goaf following excavation is 5.82280e3 KPa, which occurs on the right side wall of the third layer of the goaf. There is an area of concentrated stress with a cross-shape distribution at the location of the coal pillar. An extended area with less stress is present above the goaf.

**Figure 9:**
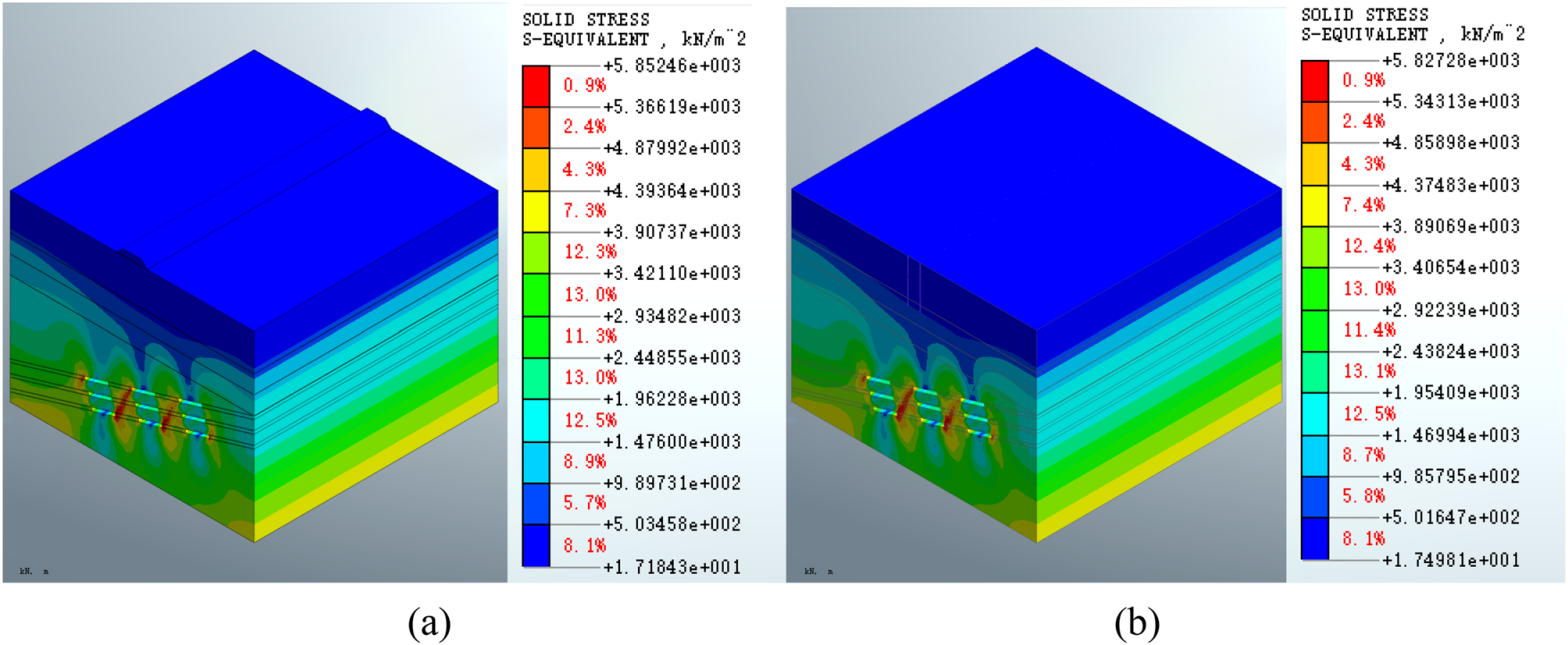
Equivalent stress on the mined-out area following construction of the surface structure for the (**a**) roadbed scheme and (**b**) pile foundation scheme.

Figure 9 shows that the maximum equivalent stress on the mined-out area increases to 5.85246e3 KPa (Fig. 9a) following the construction of the roadbed. The maximum equivalent stress on the mined-out area also increases to 5.82728e3 KPa (Fig. 9b) following pile foundation construction. The equivalent stress distribution patterns near the goaf are similar between the construction schemes. There is no significant change in the equivalent stress at the surface.

**Figure 10:**
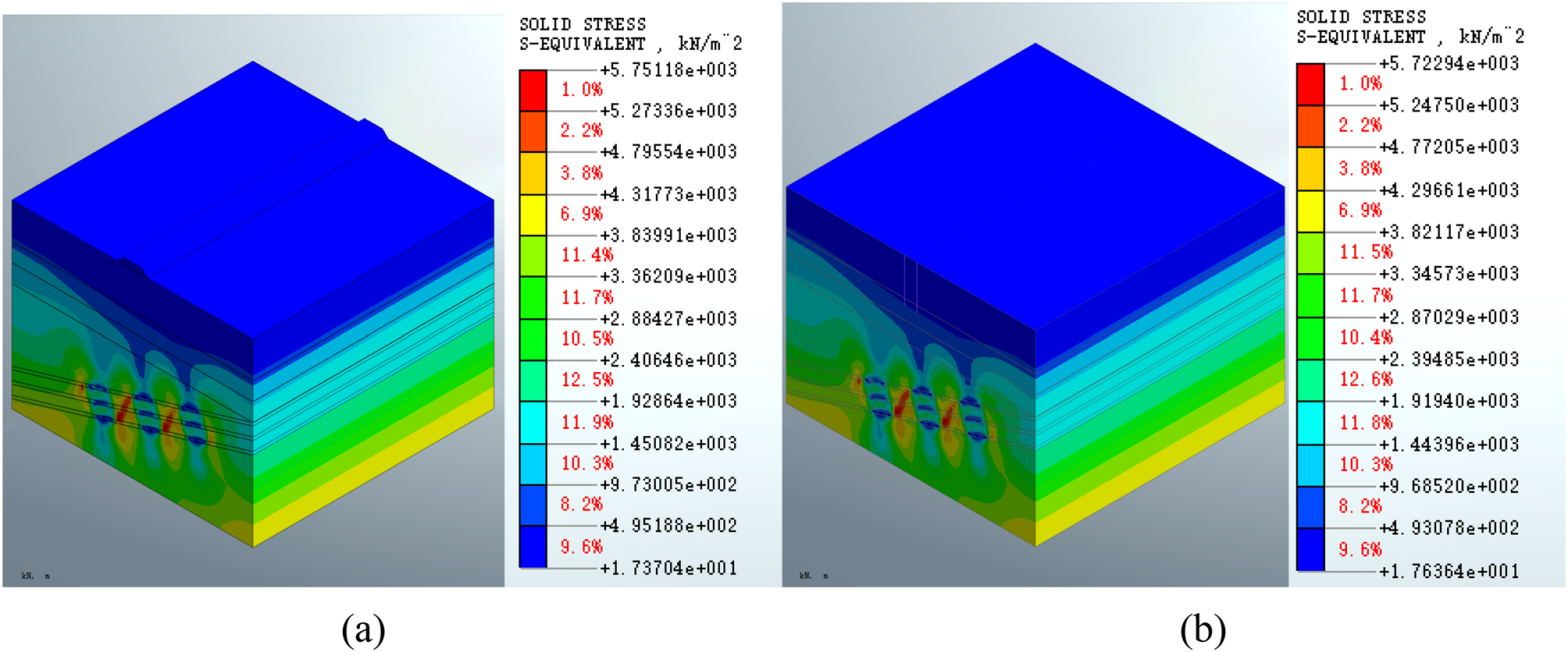
Equivalent stress on the mined-out area for the (**a**) roadbed and (**b**) pile foundation schemes following grouting treatment.

It shows that the maximum equivalent stress on the mined-out area following grouting treatment is 5.75118e3 KPa (Fig. 10a) for the roadbed scheme and 5.72294e3 Kpa (Fig. 10b) for the pile foundation scheme in Fig. 10. The maximum values are reduced compared to the pre-treatment values in both schemes. Similar equivalent stress distribution patterns present in the two schemas in the deep stratum, which is consistent with the distribution observed in the post-excavation state.

The analysis of the equivalent stress shows that the equivalent stress distribution patterns in the mined-out area formed by either the roadbed or the pile foundation schemes are similar at various stages of construction. Following surface structure construction, the maximum equivalent stress of the roadbed scheme is increased by 0.51%, and the maximum equivalent stress of the pile foundation scheme is increased by 0.08%. The maximum equivalent stress of the roadbed scheme is reduced by 1.73% post-grouting treatment, whereas the maximum equivalent stress of the pile foundation scheme is reduced by 1.79% post-treatment. Therefore, the maximum equivalent stress is reduced in the grouting treatment in both schemes. The equivalent stress index shows that the pile foundation scheme has less impact on the equivalent stress on the mined-out area.

#### Equivalent strain

**Figure 11:**
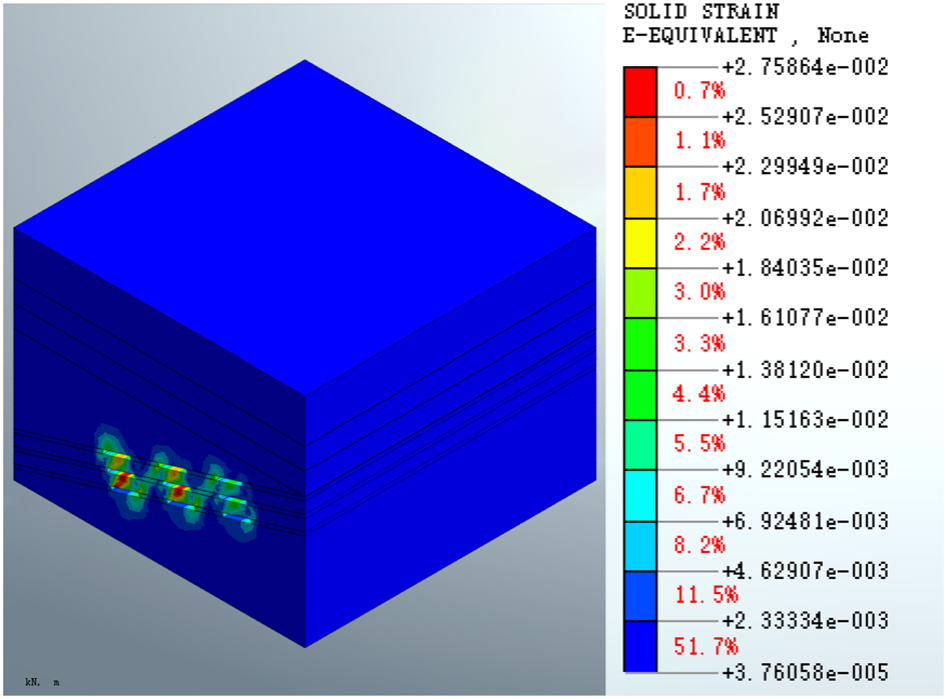
Equivalent strain of the mined-out area following excavation.

Figure 11 shows that the maximum equivalent strain on the goaf following the completion of excavation is 2.75864e − 2, which occurs in the middle strata of the goaf at the second and third layers. There is an inclined area with a continuous distribution of equivalent strain at the position of the coal pillar.

**Figure 12:**
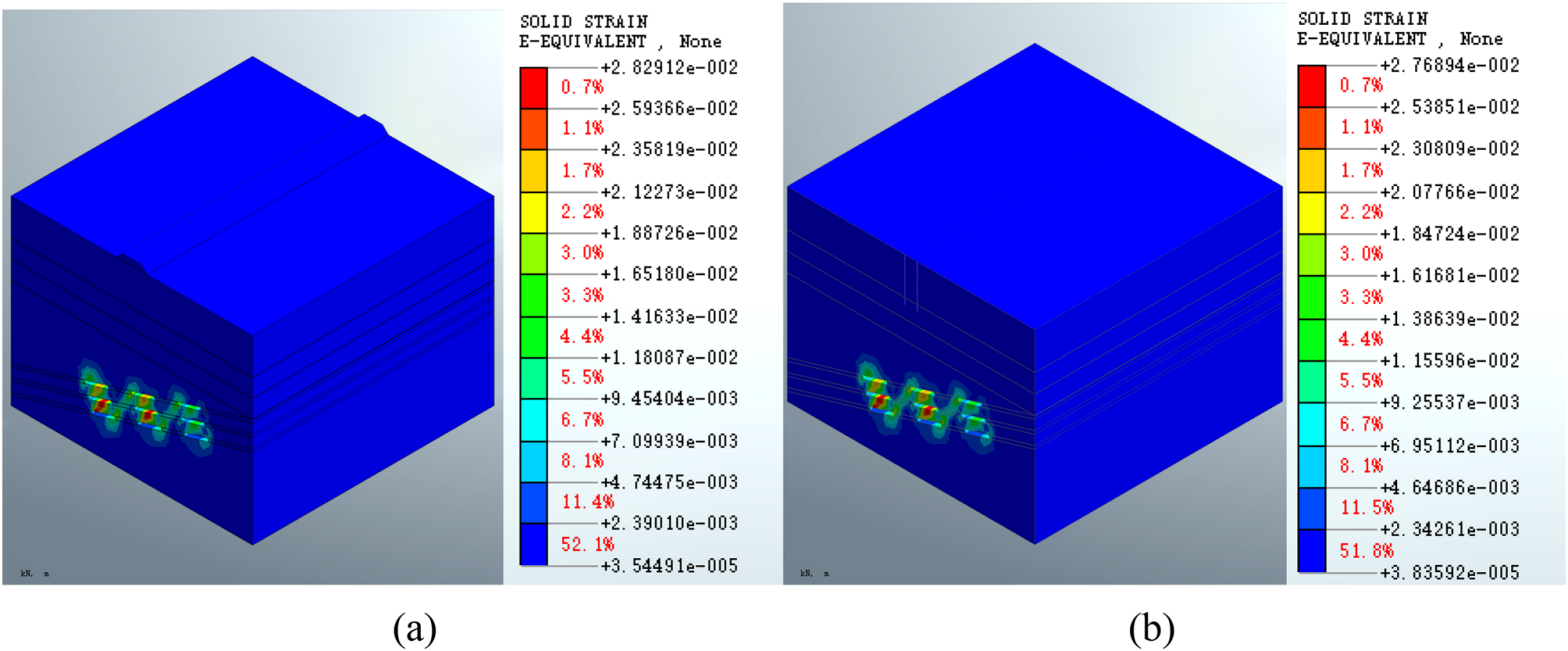
Equivalent strain on the mined-out area following (**a**) roadbed or (**b**) pile foundation construction.

Figure 12 shows that the maximum equivalent strain for the roadbed scheme is 2.82912e − 2 (Fig. 12a), which is increased compared to the post-excavation condition. In the pile foundation scheme, the maximum equivalent strain on the goaf increases to 2.76894e − 2 (Fig. 12b). The equivalent strain distribution patterns are similar. There is no significant change in the equivalent strain at the surface compared to the post-excavation condition.

**Figure 13:**
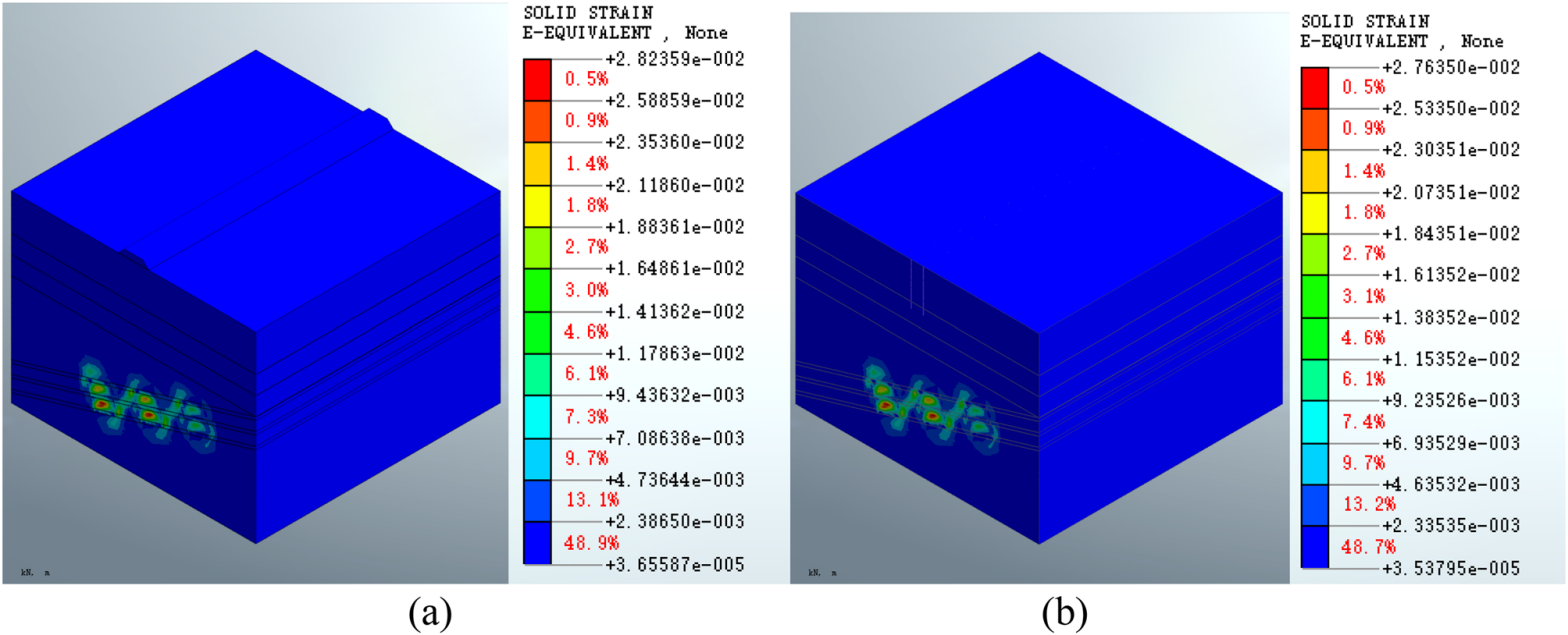
Equivalent strain on the mined-out area for the (**a**) roadbed scheme and (**b**) pile foundation scheme post-grouting treatment.

Figure 13 shows that the maximum equivalent strain on the mined-out area following grouting treatment is 2.82359e − 2 (Fig. 13a) for the roadbed scheme and 2.76350e − 2 (Fig. 13b) for the pile foundation scheme. The maximum strain is reduced in both cases compared to the post-construction condition. The two schemes have similar equivalent strain distribution patterns in the deep stratum. The equivalent strain distribution range is smaller following grouting treatment compared to the post-construction condition.

The equivalent strain results for the mined-out area show that the equivalent strain distribution patterns formed by the roadbed subgrade and pile foundation schemes are similar at various stages of construction and grouting treatment. However, the maximum equivalent strain of the roadbed subgrade scheme increased by 2.55% compared to the post-excavation condition, whereas the maximum equivalent strain of the pile foundation scheme increased by a very small percentage (approximately 0.37%). The maximum equivalent strain is reduced by 0.20% in both cases following grouting treatment. The equivalent strain index shows that the pile foundation scheme has less impact on the mined-out area. The maximum displacement, equivalent stress and equivalent strain are shown in Table 3.

**Table 3:** Maximum displacement, equivalent stress and equivalent strain for the different working conditions.

Parameter	Scheme	Post-excavation	Post-construction	Post-grouting
Displacement (m)	Roadbed	4.66980e − 1	4.81168e − 1	4.93905e − 1
	Pile foundation	4.66980e − 1	4.69107e − 1	4.81869e − 1
Equivalent stress (KPa)	Roadbed	5.82280e3	5.85246e3	5.75118e3
	Pile foundation	5.82280e3	5.82728e3	5.72294e3
Equivalent strain	Roadbed	2.75864e − 2	2.82912e − 2	2.82359e − 2
	Pile foundation	2.75864e − 2	2.76894e − 2	2.76350e − 2

#### Pile axial force

**Figure 14:**
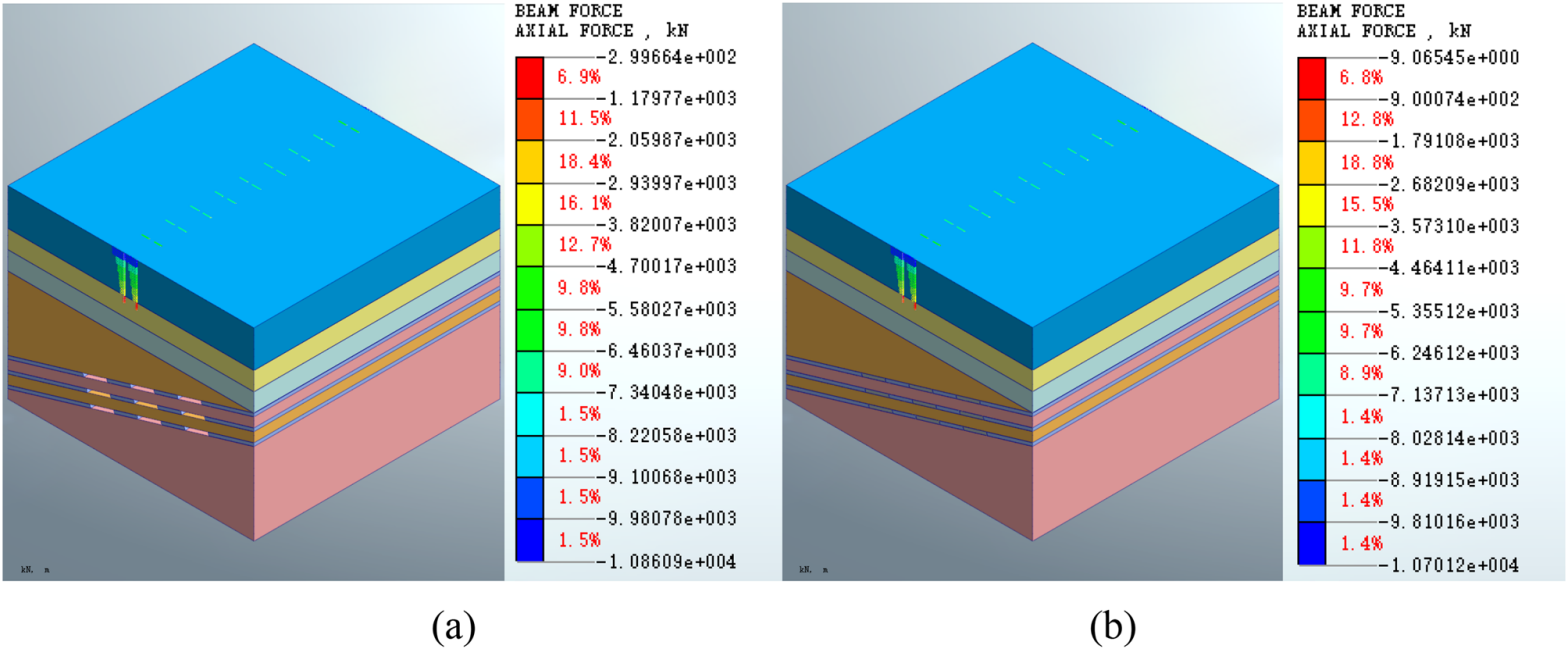
(**a**) Axial force on the pile foundation following construction (**b**) Axial force on the pile foundation following grouting treatment of the mined-out area.

Figure 14 shows that the maximum axial force on the pile foundation following its construction is − 1.08609e4 kN, and the maximum axial force on the pile foundation following grouting treatment is − 1.07012e4 kN. The maximum axial force on the pile foundation is reduced in grouting and filling the mined-out area by 1.45%.

## Conclusion

Midas/GTS NX software is used to establish a three-dimensional model of grouting treatment in the goaf of a multi-layer inclined coal seam. A new method for parameter checking based on field drilling data is proposed and used. After establishing the model, the deformation, stress and strain of the goaf under three working conditions are analyzed: goaf excavation, roadbed subgrade/pile foundation construction, and goaf grouting. It is proved that the deep-seated displacement exhibits an upward arch-type distribution pattern. The displacement compared to the post-excavation state is increased by 3.04% in case of roadbed subgrade scheme, while the increasement is 0.46% in pile foundation scheme scenario.

Stress concentration occurs at the coal pillars before and after grouting in the goaf. The concentration area is intersected. Equivalent stress is increased by 0.51% in the construction of the roadbed subgrade scheme in, while that is only 0.08% in the pile foundation scheme. After grouting treatment of the goaf under the roadbed subgrade and pile foundation schemes, the maximum equivalent stress is reduced by 1.73% and 1.79%, respectively.

A inclined continuous distribution of the equivalent strain before and after grouting treatment appeas at the position of the coal pillar. The maximum equivalent strain is increased by 2.55% in construction of the roadbed subgrade, while the increment is only 0.37% in the construction of the pile foundation. After grouting treatment, the maximum equivalent strain is reduced by 0.20% in both schemes. After grouting and filling, the maximum axial force on the pile foundation is reduced by 1.45% compared to the post-construction condition.

This research method overcomes the difficulties of validating the simulation parameters of the goaf, and can supply a good reference to follow-up goaf treatment projects in similar situations. In the mean time, the research has a strong theoretical guiding value for the study of the calculation parameters of the inclined coal seam mining-subgrade (pile foundation)-grouting system and the mechanism of force and deformation. The parameter verification and process analysis proposed in the research process are innovative in demonstration method of the foundation form of buildings or structures passing through the goaf. The influence of different foundation forms on the boundary values of the force and deformation of the goaf are focused in this research. The interrelated mechanism of the upper load and the force, as well as the deformation of the goaf and the corresponding detailed characteristics can be further explored.

## Data Availability

Some or all data, models, or code that support the findings of this study are available from the corresponding author upon reasonable request.
